# Effects of biochar on soil properties as well as available and TCLP-extractable Cu contents: a global meta-analysis

**DOI:** 10.1038/s41598-025-18170-z

**Published:** 2025-09-25

**Authors:** Xiaowen Teng, Dong Huang, Yuyou Zhi, Yaqian Li, Dubin Dong, Xuqiao Wu, Yini Wang, Zhoujia Jiang, Hao Huang, Yanxin Tang, Dan Liu, Weijie Xu

**Affiliations:** 1 State Key Laboratory of Subtropical Silviculture, Key Laboratory of Soil Remediation and Quality Improvement of Zhejiang Province, Zhejiang A&F University, Lin’an, 311300 China; 2 Pujiang County Ecological Civilization Promotion Center, Jinhua, 322200 China; 3 Yanguan Town People’s Government, Jiaxing, 323500 China; 4 College of Life Science and Technology, Central South University of Forestry and Technology, Changsha, 410004 China

**Keywords:** Copper, Amendment, Soil, biochar characteristics, Meta-analysis, Impact, Biogeochemistry, Ecology, Environmental sciences

## Abstract

Biochar is an eco-friendly soil amendment that has been demonstrated to have significant potential for regulating soil properties and immobilizing heavy metals. In this study, a meta-analysis was performed to synthesize global data from 41 peer-reviewed studies (2012–2024) and evaluate the effects of biochar on soil chemistry and copper (Cu) mobility. The results revealed that biochar application increased the soil pH, cation exchange capacity (CEC), and electrical conductivity (EC) while decreasing the available and TCLP-extractable Cu contents. Biochar with an ash content > 60% elevated the soil pH by ≥ 32.4%, and biochar with a specific surface area (SSA) of 50–100 m²/g enhanced the CEC by ≥ 183%. Low nitrogen content (N% <2%) increased EC by ≥ 61.27%, whereas high pH (> 7.5) and oxygen content (O% >20%) significantly reduced the available Cu (≥ 37.72% and ≥ 22.31%, respectively) and TCLP-extractable Cu (≥ 34.97% and ≥ 24.07%, respectively) contents. Notably, the largest improvement in pH occurred in highly acidic soils (initial pH = 3), and biochar with a pH > 7.5 most effectively reduced Cu mobility. Notably, the ability of biochar to immobilize Cu was independent of the initial soil pH. The immobilization of copper by biochar primarily involved several mechanisms, including precipitation, surface complexation with oxygen-containing functional groups, ion exchange, and physical adsorption within its porous structure. These findings provide actionable insights for the sustainable management of soil by highlighting the ability of biochar to increase soil fertility and remediate Cu-contaminated soils through chelation with Cu functional groups.

**Supplementary Information:**

The online version contains supplementary material available at 10.1038/s41598-025-18170-z.

## Introduction

Agricultural productivity is increasingly constrained by progressive soil degradation and inefficient nutrient utilization, exacerbating challenges in food security worldwide^1^. Soil that is contaminated with potentially toxic elements (PTEs), such as those introduced by mining, overfertilization, and poor sewage irrigation, poses a critical threat to agricultural sustainability and ecological security worldwide^2–4^. According to a nationwide survey in China, the total area of arable land contaminated by heavy metals is approximately 20 million hectares, accounting for 16.1%of all arable land^5^. Copper (Cu), which is predominantly found in mineral forms such as chalcopyrite (Cu_2_S), chalcopyrite (CuFeS_2_), and malachite (Cu_2_(OH)_2_CO_3_)^6^becomes unstable under aerobic and hydrous conditions, releasing divalent cations (Cu^2+^) into the soil. Cu is the third-largest source of heavy metal pollution in China, following only arsenic and cadmium^7^. While Cu is an essential micronutrient, excessive soil concentrations could severely threaten plant growth^8^. Methods for the remediation of soil contaminated with Cu include in situ restoration and ectopic restoration^9^. In situ stabilization has been considered an effective approach to remediate Cu-contaminated soil because of its cost-effectiveness and convenient application^10,11^. Numerous amendments, including silica, zeolite lime, phosphate, compost, fertilizer, hydroxyapatites and bentonite, have been applied to improve soil quality by reducing the bioavailability and leaching of Cu in soil^12–15^. Biochar, which is a porous carbonaceous material, has been proposed as tool for carbon sequestration and soil amendment^16,17^. Previous studies have reported that the chemical properties of soil, such as the pH, cation exchange capacity (CEC), electrical conductivity (EC), and extractable nutrients of soil, can be increased to certain extents via a manner that mainly depends on different types of biochar^18–21^.

However, previous research on the effects of biochar application on the remediation of soils contaminated by heavy metals (especially Cu) is not conclusive. As a quantitative review, a meta-analysis could address this issue by calculating the overall effect sizes and summarizing the results of the main articles to reveal sources of variation across studies^22^. In recent years, the number of meta-analyses have quantitatively assessed the impacts of biochar on the remediation of soil contamination and found that application led to a continuous improvement in soil quality. Studies have shown that soil pH is the most important factor that affects the changes in heavy metals in biochar-amended soils, followed by soil texture, aging time, and pyrolysis temperature of biochar^23^. The impacts of biochar on the chemical properties of soil depend mainly on the biochar application rate, initial soil pH, and sand content in the soil^24^. The application of biochar can increase the pH, CEC, and organic carbon content of soil by 46%, 20%, and 27%, respectively^25^. Additionally, adding biochar to the soil can reduce the accumulation of Cu in plant tissues by an average of 25%. However, the effect on plant Cu concentrations is more significant when biochar is applied to alkaline soils^26^. However, some studies suggest that the potential of biochar to reduce metal toxicity is more strongly driven by the soil system than the plant system^27^. Despite the growing interest in biochar-based remediation, previous studies have yielded inconsistent findings about its effects on the chemical properties and Cu bioavailability of soil under different environmental and material conditions. These inconsistencies are primarily attributed to variations in biochar feedstocks, pyrolysis conditions, application rates, and soil characteristics, which complicate the generalization of conclusions across studies. While individual experiments often focus on isolated soil parameters or site-specific results, a comprehensive, quantitative synthesis that links biochar physicochemical traits with the chemical responses and Cu mobility of soil on a global scale is still lacking. Therefore, a meta-analytical approach is warranted to systematically identify patterns, quantify overall effects, and elucidate the mechanisms underlying these interactions. This study involved such an assessment, offering evidence-based insights to support the design and application of more effective biochar amendments in Cu-contaminated soils.

The aim of this study was to address these knowledge gaps by conducting a global meta-analysis to understand how biochar and its physicochemical properties affect changes in the chemical properties and Cu bioavailability of soil. With these considerations in mind, a meta-analysis of 41 articles was performed with the following objectives: (1) to quantify the impact of biochar physicochemical properties on the chemical properties and copper bioavailability of soil and (2) to assess the combined effects of biochar on the chemical properties of soil and the bioavailability of Cu in soil.

## Data and methods

### Data compilation

The data for this study were collected from peer-reviewed literature retrieved from the Web of Science (WOS) and China National Knowledge Infrastructure (CNKI) using the following terms: “biochar”, “Cu pollution”, “Cu”, and “soil remediation” (Fig. S1). A total of 725 articles published between 2012 and 2024 were identified, among which 41 met the following selection criterion:

Soil and experimental conditions: Articles must specify soil type, experimental duration, and environmental conditions;

*Experimental design* Studies had well-defined treatment and control groups with biochar application as the sole experimental variable;

*Measured parameters* pH, electrical conductivity (EC), cation exchange capacity (CEC), available Cu, and Toxicity Characteristic Leaching Procedure (TCLP), as well as Cu content, were reported for both treatment and control groups before and after biochar application.

*Replicates* Treatments and controls included at least three replicates;

*Data presentation* Results were expressed as the means ± standard deviations (SDs) or standard errors (SEs);

*Quality control* Studies demonstrated analytical quality assurance protocols to ensure reliability.

The soil and biochar properties obtained from the selected articles were analyzed. The soil properties included the pH, electrical conductivity (EC), cation exchange capacity (CEC), and concentrations of available Cu and TCLP-extractable Cu. The available Cu concentrations were standardized to mg/kg when reported in alternate units. For previous studies that presented fractions in absolute concentrations, these percentages were manually calculated relative to the total concentration. The biochar properties included pH, ash content, specific surface area (SSA), CEC, EC and elemental composition (C%, H%, O%, and N%). All the variables and their definitions are listed in Table 1.

**Table 1:** Classification of indicators and subgroup variables.

Indicators	Classification	Remarks
Soil	Acidic soil	PH < 6.5
	Non-acidic soil	PH > 6.5
Physicochemical properties of biochar	PH	6.5 ~ 7.5, > 7.5
	Ash content	< 20%, 20 ~ 40%, 40 ~ 60%, > 60%
	Specific surface area (SSA)	< 50 m^2^/g, 50 ~ 100 m^2^/g, > 100 m^2^/g
	Cation exchange capacity (CEC)	< 50 cmol^+^/kg, 50 ~ 100 cmol^+^/kg, > 100 cmol^+^/kg
	Electrical conductance (EC)	< 0.48 S/m, 0.8 ~ 1.6 S/m,>1.6 S/m
	Percentage of carbon (C%)	< 30%, 30 ~ 60%, > 60%
	Percentage of hydrogen (H%)	< 2%, 2 ~ 4%, > 4%
	Percentage of oxygen (O%)	< 10%, 10 ~ 20%, > 20%
	Percentage of nitrogen (N%)	< 2%, 2 ~ 4%, > 4%

### Database construction

The data comprised 593 observations from 41 scientific articles (Text S1) and were divided into biochar treatment and control groups. According to the data, 68% (*n* = 408) of the observations were from acidic soils, while 32% (*n* = 185) were from nonacidic soils. Data was digitized from published sources and presented graphically using GetData version 2.26 (GetData Pty Ltd., USA). The effects of biochar application were represented in terms of effects on the soil pH, electrical conductivity, cation exchange capacity, available Cu concentration, and concentration of TCLP-extractable Cu. Soil pH values extracted by KCl and CaCl_2_ were converted to deionized water-extractable pH values by Eqs. (1) and (2)^28^. SD values for every treatment and control group in the reviewed studies were recorded. If an SE value was reported in the article, it was converted to an SD value using Eq. (3) to ensure a consistent statistical standard.

### Meta-analysis

The meta-analysis was conducted in a two-stage approach. First, the impacts of biochar on the soil pH, EC, CEC, available Cu contents, and TCLP-extractable Cu contents were evaluated. Second, the combined effects of biochar physicochemical properties on these variables were assessed. In a sub-meta-analysis, the mean effect sizes for the biochar groups across the soil pH categories were calculated. Effect sizes were determined using the natural log of the response ratio (ln RR), a standard meta-analytic metric^29^:

1 $$PH\left( {H_{2} O} \right) = PH\left( {KC{\text{l}}} \right) \times 0.74 + 1.96$$

2 $$PH\left( {H_{2} O} \right) = PH\left( {{\text{CaCl}}_{{\text{2}}} } \right) \times 0.8{\text{6}} + 1.6{\text{5}}$$

3 $${\text{SD}} = SE\sqrt {\text{n}}$$

4 $${\text{ln (}}RR) = {\text{ln}}\frac{{X_{{\text{t}}} }}{{X_{{\text{c}}} }}$$

where Xt and Xc are the means of the variables in the biochar treatment and control groups, respectively. The effect sizes were weighted by the inverse of the pooled variance (v) using Eq. (5).

5 $${\text{v}} = \frac{{S_{{\text{t}}} ^{2} }}{{{\text{n}}_{{\text{t}}} {\text{X}}_{{\text{t}}} ^{{\text{2}}} }} + \frac{{S_{{\text{c}}} ^{2} }}{{{\text{n}}_{{\text{c}}} {\text{X}}_{{\text{c}}} ^{{\text{2}}} }}$$

where nt and nc are the sample sizes for the treatment and control groups, respectively, and st and sc are the SDs for the treatment and control groups, respectively. The weighted mean response ratio (ln RR++) was calculated using Eq. (6)

6 $${\text{ln (}}RR_{{ + + }} )\frac{{\sum\nolimits_{{i = 1}}^{{\text{m}}} {\sum\nolimits_{{{\text{j}} = {\text{1}}}}^{{\text{k}}} {{\text{Wij(ln(RR))}}} } }}{{\sum\nolimits_{{i = 1}}^{{\text{m}}} {\sum\nolimits_{{j = 1}}^{k} {Wij} } }}$$

where the weighting of each response ratio (w) is the reciprocal of its variance {Citation}, m is the number of groups, and k is the number of observations in the ith group. The results of the ln RR analyses were back-transformed and reported as the percentage change in the indicator to facilitate interpretation. The percentage change in the indicator was calculated using Eq. (7)^28^:

7 $${\text{Percentage change}} = {\text{exp}}[\ln (RR_{{ + + }} )] \times 100{\text{\% }} - 100\%$$

### Data analysis

All the statistical analyses were performed using R (version 3.6.0) software. The mean effect sizes were calculated using the mixed-effects models of the R package “metafor” to calculate the average effect size and converted into a 95% confidence interval (CI)^30^. When the 95% CI did not overlap with zero, biochar addition was considered to have significantly affected the indicator. The relationships of the ln RR of the indicators (available Cu content, TCLP-extractable Cu content, EC, and CEC) with the ln RR of the soil pH, the original pH of the soil and the physicochemical properties of the biochar (pH, Ach content, SSA, CEC, EC, and elemental composition) were determined using regression analysis. A regression analysis was performed to examine the pairwise relationships between the natural logarithms of the response ratios of the indicators. We used regression fitting functions (linear, quadratic, exponential rise or decay, and exponential growth equations) appropriate for (e.g., best R^2^ values and greatest p values) the presented data. Linear regression analyses were conducted using the R package “ggplot2”^31^. The assessments of publication bias were completed with Rosenberg’s method (Table S1). Additionally, Rosenberg’s fail-safe number was calculated^32^. A fail-safe number is usually considered robust if it is > 5n + 10, where n is the original number of studies^33^.

## Results

### Impact of biochar application on the chemical properties of soil

**Fig. 1:**
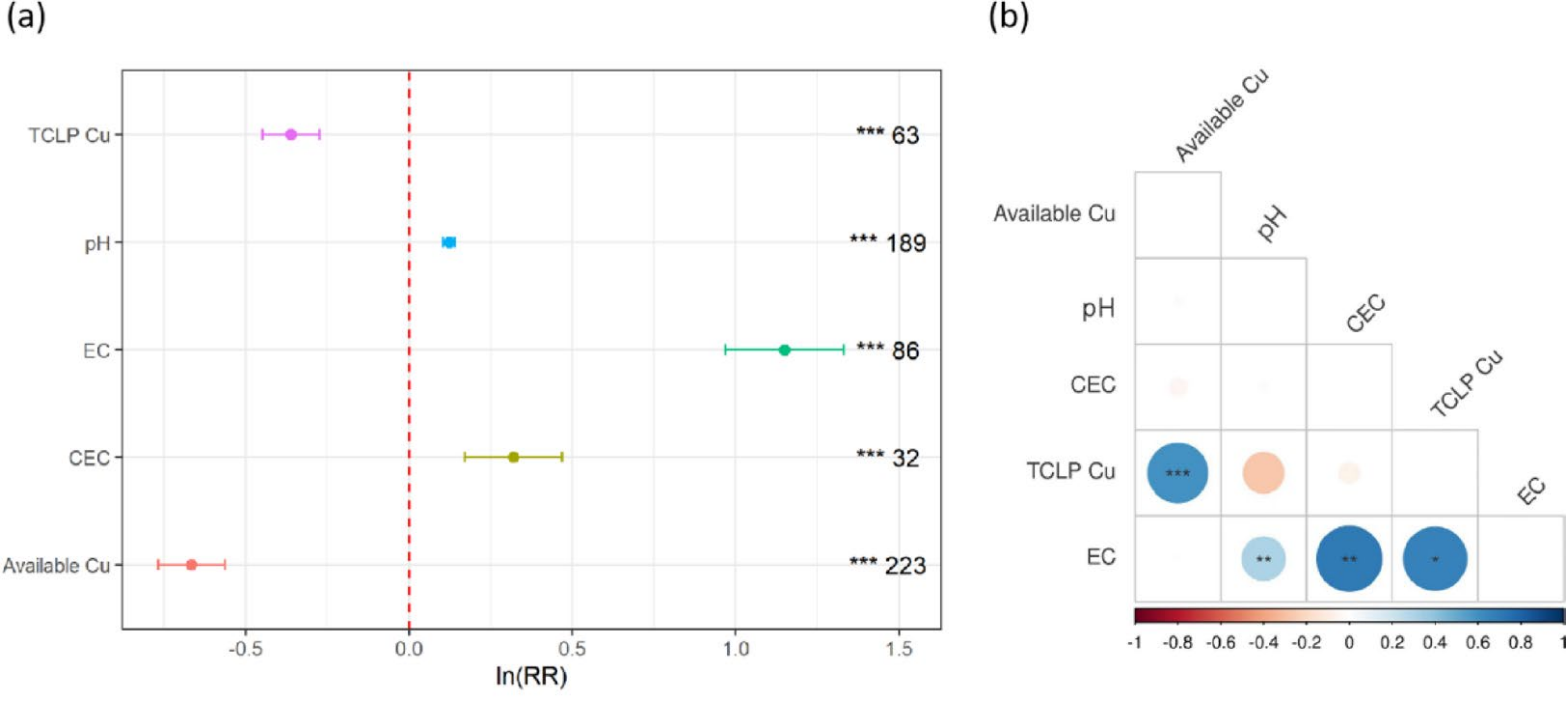
(**a**) Overall impact of biochar application on soil pH, EC, CEC, TCLP-extractable Cu, and available Cu. (**b**) Regression analysis of the response ratios of soil pH, EC, CEC, TCLP-extractable Cu, and available Cu to the natural logarithmic relationship. The x-axis represents the response ratio of each indicator. In the regression analysis, circles represent the R² values, with larger circles indicating higher R². Orange circles indicate negative correlations, while blue circles indicate positive correlations. The *p*-values for different responses are indicated by “*” and “NS” (“*” denotes 0.01 < *p* < 0.05; “**” denotes *p* < 0.01; “***” denotes *p* < 0.001; “NS” denotes *p* ≥ 0.05). The Arabic numerals on the right represent the number of observations.

Overall, compared with the control, biochar application significantly affected the pH, EC, and CEC of the soil (*P* < 0.001, Fig. 1a). To evaluate the impact of biochar on these parameters across soil types, we classified the soils as acidic (pH < 6.5) and nonacidic (pH > 6.5) soils based on their initial pH. Compared with those of the control group, the pH, EC and CEC of soil significantly increased by 12.6%, 70.30% and 26%, respectively, after biochar application. The soil pH significantly increased across all soil types (*P* < 0.001), with the most pronounced effect (66%) observed in acidic soils. Similarly, CEC enhancement was significantly greater in acidic soils (*P* < 0.001) than in nonacidic soils (*P* < 0.01; Fig. 3). The EC exhibited a universal increase (*P* < 0.001).

**Fig. 2:**
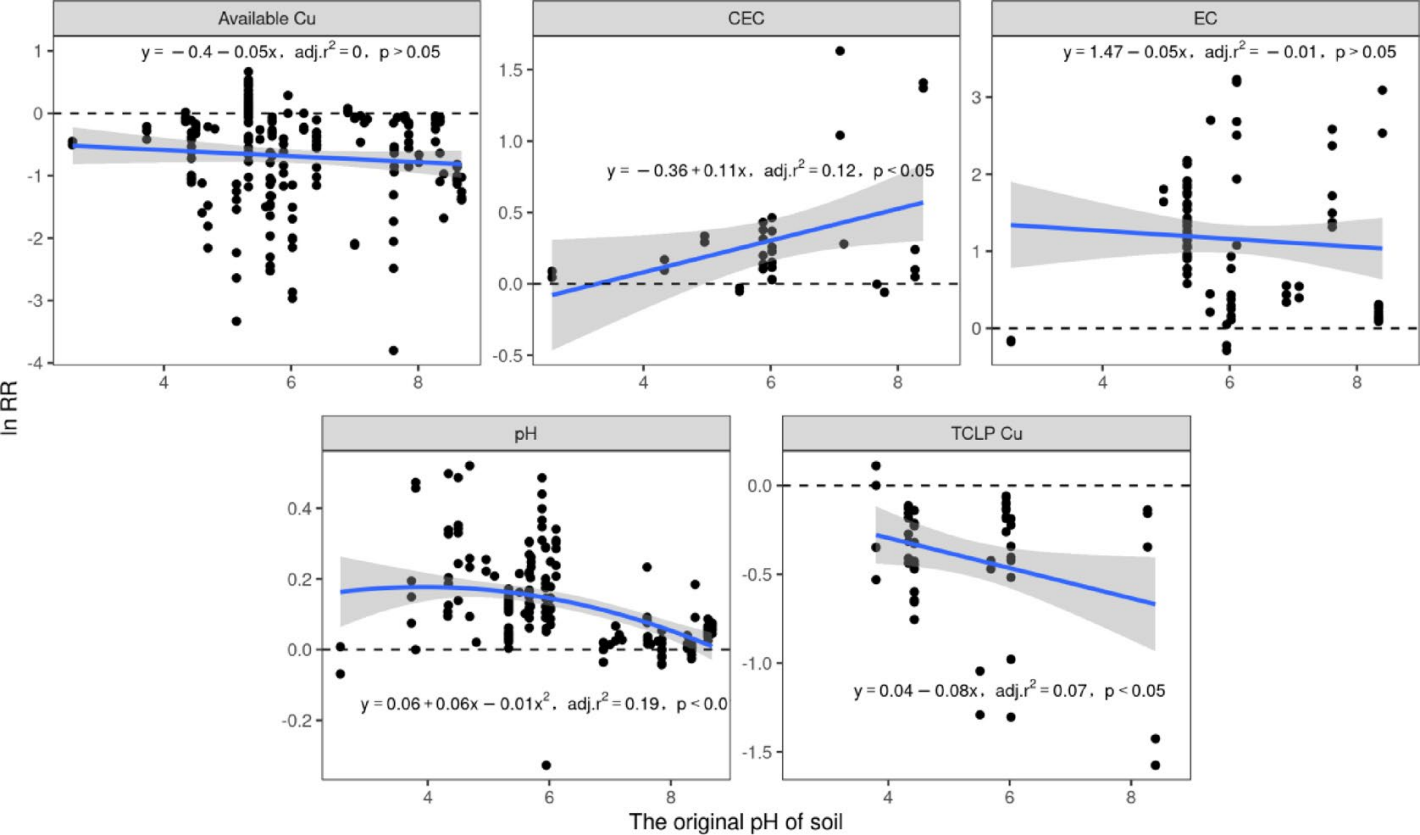
Regression analysis of the relationship between the original soil pH and soil pH, EC, CEC, TCLP-extractable Cu, and available Cu. The x-axis represents the original soil pH, and the y-axis shows the response ratios of soil pH, EC, CEC, TCLP-extractable Cu, and available Cu. The shaded area represents the 95% confidence interval.

Regression analysis revealed a weak positive correlation between the pH and EC of the control soil (R² = 0.10, *P* < 0.01, Fig. 1b) but a stronger positive correlation in both acidic soils (R² = 0.24, *P* < 0.01) and nonacidic soils (R² = 0.74, *P* < 0.01) (Fig. 4). These findings indicate that pH and EC increase concurrently in biochar-treated soil. In acidic soils, pH was positively correlated with CEC (R² = 0.28, *P* < 0.01), whereas the CEC and EC were moderately positively related (R² = 0.49, *P* < 0.01) (Fig. 2).

The regression analysis demonstrated that the initial soil pH positively predicted biochar-induced CEC enhancement (response ratio “RR”, R² = 0.12, *P* < 0.05), which suggested greater improvement in the CEC in less acidic soils. Conversely, initial pH was negatively correlated with pH change (RR, R² = 0.19, *P* < 0.01), indicating diminished pH buffering in higher-pH soils. Biochar had the greatest effect on soil with an initial pH value of 3.

### Impact of biochar on the available Cu and TCLP-extractable Cu contents of soil

**Fig. 3:**
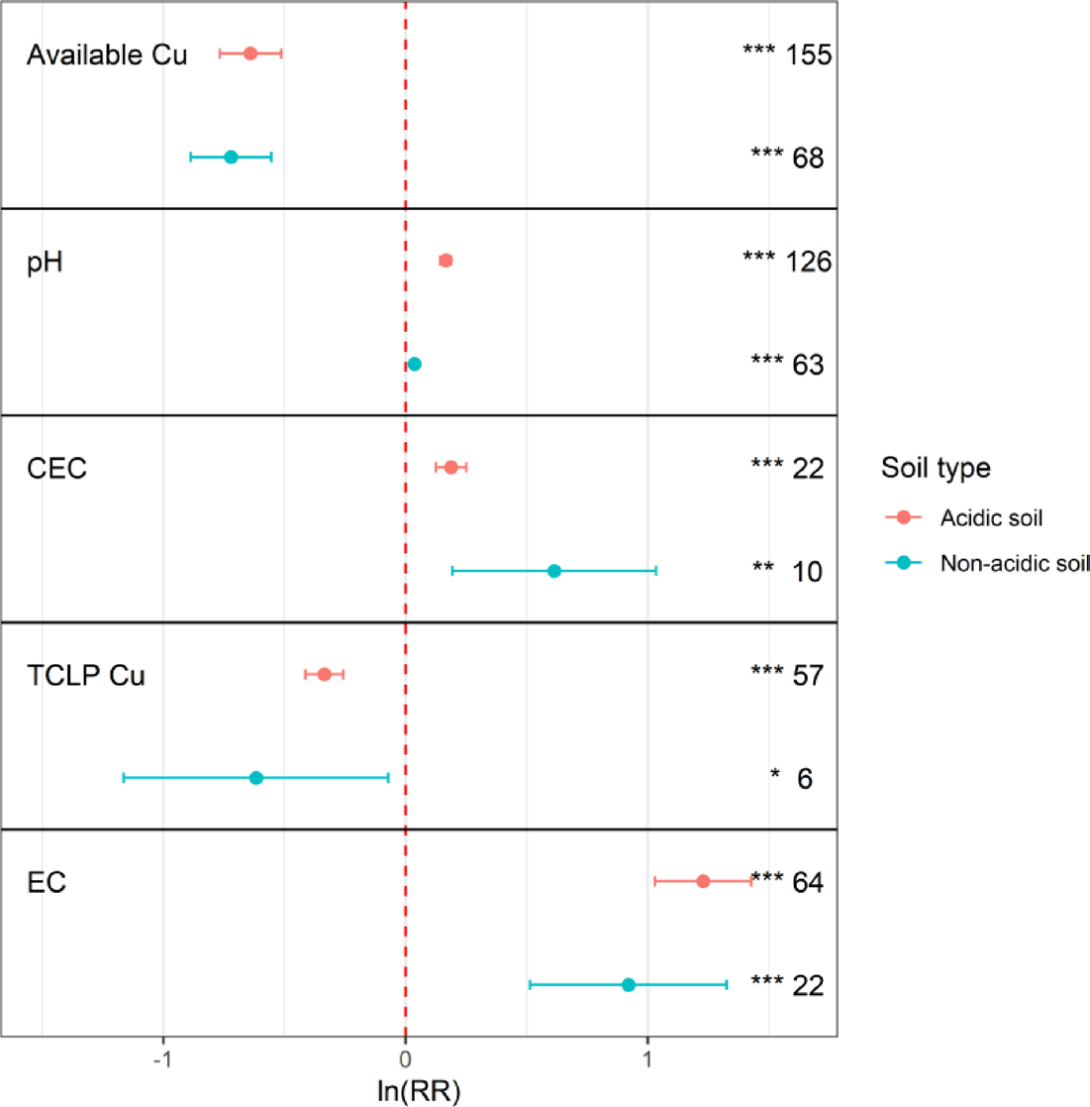
Impact of biochar application on pH, EC, CEC, TCLP-extractable Cu, and available Cu in acidic and non-acidic soils. The x-axis represents the response ratios of each indicator. The *p*-values for different responses are indicated by “*” and “NS” (“*” denotes 0.01 < *p* < 0.05; “**” denotes *p* < 0.01; “***” denotes *p* < 0.001; “NS” denotes no statistical significance, *p* ≥ 0.05). The Arabic numerals on the right represent the number of observations.

Biochar application significantly affected the available Cu and TCLP-extractable Cu contents in the soil. After biochar application, the concentrations of available Cu and TCLP-extractable Cu were generally 34.35% and 30.97% lower than those in the control soil. The reduction in available Cu was significant across all soil types (*P* < 0.001), with the greatest reduction observed in acidic soils (69%) (Fig. 3). The decrease in the TCLP-extractable Cu concentration in acidic soils was significantly greater (*P* < 0.001) than that in nonacidic soils (*P* < 0.05). According to the regression analysis between various indicators (Fig. 1b), available Cu and TCLP-extractable Cu contents in the soil were strongly positively correlated (R² = 0.34, *P* < 0.001), indicating that the CEC and EC of soil were moderately positively related after biochar application.

**Fig. 4:**
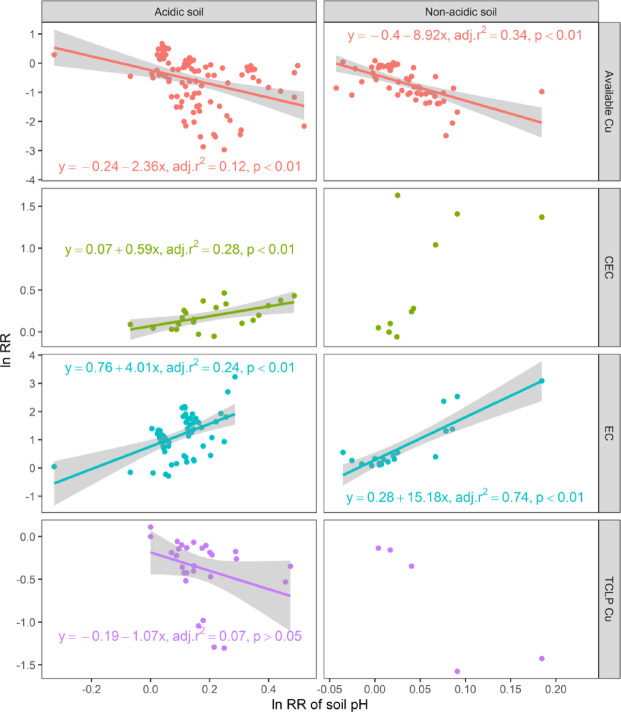
Regression analysis of the relationship between soil pH and EC, CEC, TCLP-extractable Cu, and available Cu in acidic and non-acidic soils. The x-axis represents the response ratio of soil pH, while the y-axis represents the response ratios of EC, CEC, TCLP-extractable Cu, and available Cu.

Regression analysis revealed a significant negative correlation between changes in the pH and available copper (Cu) concentrations of soil (Fig. 4, *P* < 0.001). Specifically, in acidic soils (R² = 0.12) and neutral soils (R² = 0.34, *P* < 0.001), elevated pH levels corresponded with reduced available Cu. Additionally, initial soil pH (prior to biochar application) negatively influenced the response ratio of TCLP-extractable Cu (R² = 0.07, *P* < 0.05), indicating that the efficacy of biochar in immobilizing TCLP-extractable Cu contents improved in soils with a higher baseline pH. These findings highlight the pH-dependent variability in the effects of biochar on TCLP-extractable Cu contents. A significant relationship was not found between initial soil pH and available Cu reduction (*P* > 0.05), demonstrating that biochar-mediated decreases in available Cu contents are independent of original soil pH conditions.

### Impact of biochar physicochemical properties on soil chemical properties

**Fig. 5:**
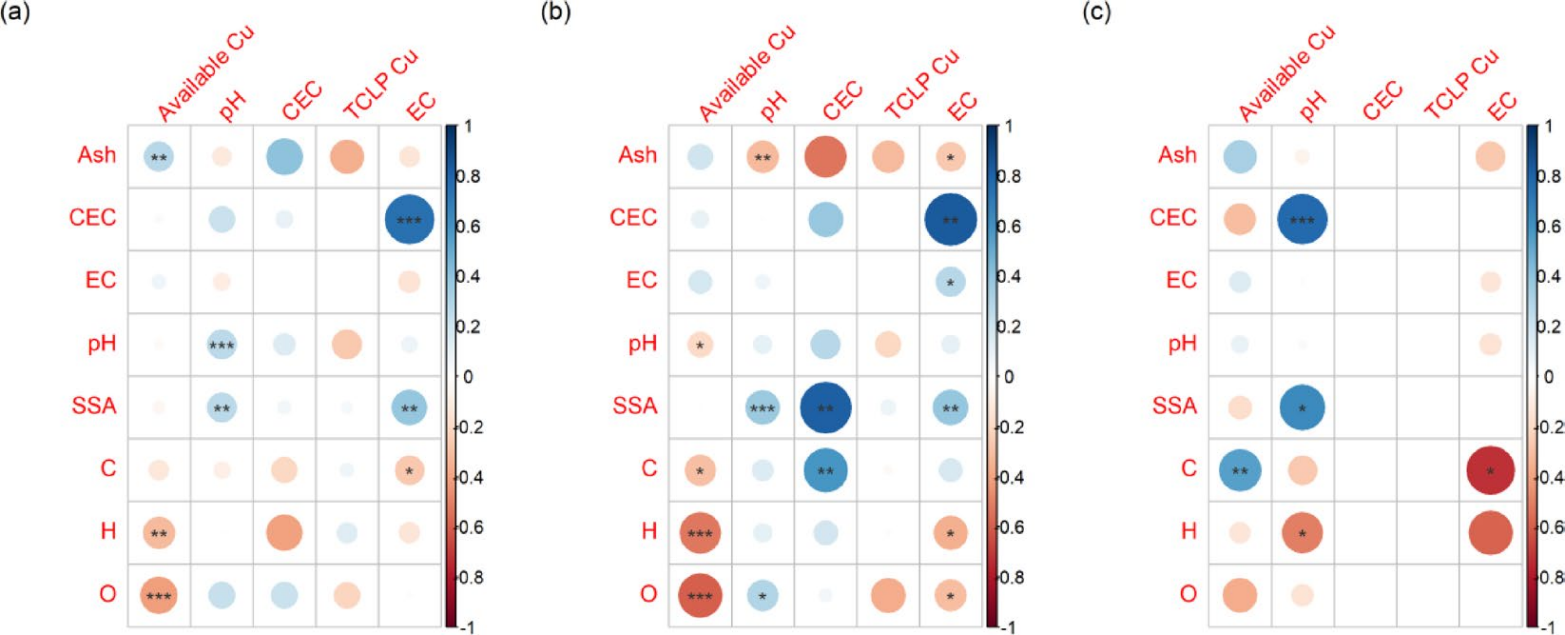
Regression analysis of the relationship between biochar physicochemical properties and the response ratios of soil pH, EC, CEC, TCLP-extractable Cu, and available Cu in soils of different pH levels. (**a**) Overall effect; (**b**) acidic soils; (**c**) non-acidic soils. In the regression analysis, circles represent R² values, with larger circles indicating higher R² values. Red circles indicate a negative correlation, while blue circles indicate a positive correlation. The *p*-values for different responses are indicated by “*” and “NS” (“*” denotes 0.01 < *p* < 0.05; “**” denotes *p* < 0.01; “***” denotes *p* < 0.001; “NS” denotes *p* > 0.05).

The pH, CEC, and EC of soil were influenced by the properties of the biochar (Fig. 5). Overall, soil pH was significantly positively correlated with biochar pH (R² = 0.0053, *P* < 0.001). A positive correlation between soil pH and the biochar SSA (R² = 0.037, *P* < 0.01) (Fig. 5a), with a strong correlation coefficient, was more observed in acidic soils (R² = 0.75, *P* < 0.001) than in nonacidic soils (R² = 0.35, *P* < 0.05). In nonacidic soils, soil pH was positively correlated with the CEC of biochar (R² = 0.59, *P* < 0.001) (Fig. 5c). For acidic soils, the CEC was positively correlated with both the biochar SSA and C content (R² = 0.65, *P* < 0.01 and R² = 0.34, *P* < 0.01) (Fig. 5b). When soil pH was excluded as a variable, the EC of soil was positively correlated with both the CEC and SSA of biochar (R² = 0.94, *P* < 0.001 and R² = 0.067, *P* < 0.01) but negatively correlated with C content (R² = 0.057, *P* < 0.05) (Fig. 5a). In acidic soils, biochar CEC and SSA are positively correlated with the EC of soil (R² = 0.69, *P* < 0.01 and R² = 0.14, *P* < 0.01), whereas in nonacidic soils, the EC of soil is negatively correlated with the C content of biochar (R² = 0.028, *P* < 0.05) (Fig. 5c).

**Fig. 6:**
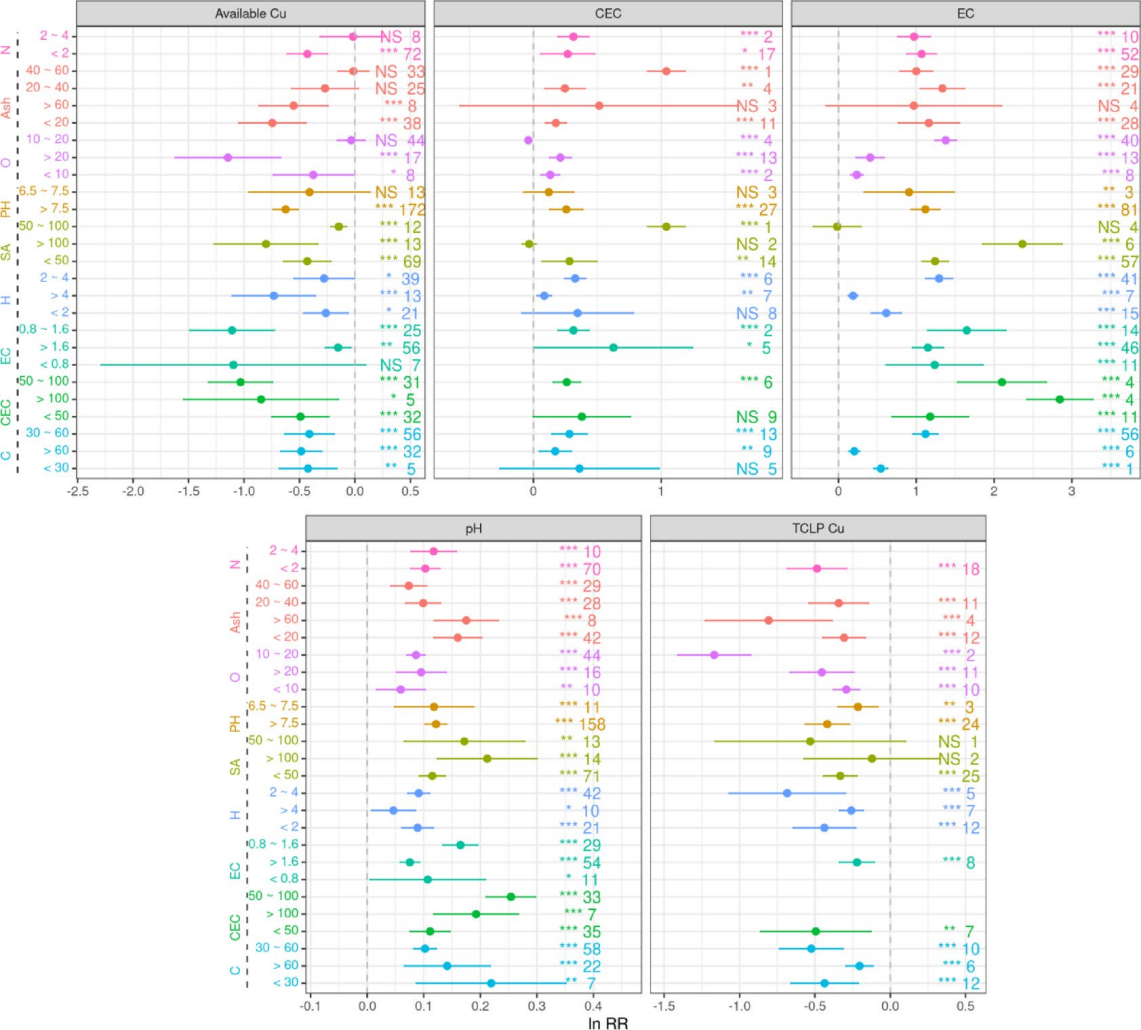
The impact of biochar physicochemical properties on soil pH, EC, CEC, TCLP-extractable Cu, and available Cu. The x-axis represents the response ratios of soil pH, EC, CEC, TCLP-extractable Cu, and available Cu. The y-axis represents the corresponding groups of biochar categorized by physicochemical properties. Arabic numerals indicate the number of observations. The *p*-values for different responses are denoted by “*” and “NS” (“” denotes 0.01 < *p* < 0.05; “” denotes *p* < 0.01; “*” denotes *p* < 0.001; “NS” denotes *p* ≥ 0.05).

We further quantified the effects of the physicochemical properties of biochar on the pH, CEC and EC of soil (Fig. 6). Compared with the control, biochar with an ash content greater than 60% had the most pronounced effect, increasing the soil pH by 20.78%. Similarly, a biochar oxygen content greater than 20% resulted in a 9.3% increase in soil pH compared to that of the control. Biochar with a 2–4% hydrogen (H) content significantly increased soil pH by 9.8% in comparison to the control. An elevated oxygen content (> 20%) enhanced the CEC of soil by 27.23% compared to the control. The biochar with SSA values of 50–100 m²/g significantly increased the CEC of soil by 183%, and an ash content of 20–40% significantly enhanced the EC of soil by 112%, compared to the control. Additionally, a biochar nitrogen (N) content of less than 2% increased the EC of soil by 61.27% in comparison to the control.

### Impact of biochar physicochemical properties on soil available Cu and TCLP-extractable Cu contents

The availability of Cu and TCLP-extractable Cu is influenced by the properties of biochar (Fig. 5). When soil pH was not considered, the available Cu content in soil exhibited a positive correlation with the ash content in biochar (R² = 0.30, *P* < 0.01) and negative correlations with biochar O and H contents (R² = 0.13, *P* < 0.001 and R² = 0.022, *P* < 0.01) (Fig. 5a). In acidic soils, the available Cu content in soil was negatively correlated with the O and H contents in biochar (R² = 0.34, *P* < 0.001 and R² = 0.27, *P* < 0.001) (Fig. 5b). For nonacidic soils, the available Cu content in soil was positively correlated with the C content in biochar (R² = 0.29, *P* < 0.01) (Fig. 5c).

The effects of biochar properties on the available Cu and TCLP-extractable Cu contents in soil are shown in Fig. 6. Biochar with a pH greater than 7.5 resulted in the most pronounced reduction in available Cu (37.72% decrease relative to the control) and TCLP-extractable Cu (34.97% decrease relative to the control) contents. Similarly, when the O and H contents in biochar exceeded 20% and 4%, the available Cu content decreased by 22.31% and 21.59% and the TCLP-extractable Cu content decreased by 24.07% and 44.98%, respectively, compared with those of the control.

## Discussion

### pH elevation mechanisms

Our results show that biochar has a significant effect on the chemical properties of soil^34,35^. The results of the meta-analysis are consistent with these studies. The application of biochar significantly increased soil pH (*P* < 0.001). According to the literature, several studies have indicated that biochar application can increase the soil pH through various mechanisms. First, biochar can regulate soil pH due to its alkalinity and high pH buffering capacity^35,36^. Biochar generally contains salt-based ions, such as K^+^, Ca^2+^, and Mg^2+^, that are released exchanged with Al^3+^ and H^+^ when biochar is applied to the soil. These phenomena lead to a decrease in H^+^ levels and an increase in pH of soil^37,38^. Additionally, we speculate that the unique pore structure of biochar can adsorb air, water, and inorganic nutrients, thereby providing a suitable habitat for microorganisms to survive and flourish, and that changes in the abundance and community structure of microorganisms indirectly affect the soil pH^39^. Furthermore, the carbonates and oxides that are present in biochar, particularly in high-temperature pyrolyzed materials, also contribute to pH elevation by neutralizing soil acidity^12^. The results of the current meta-analysis also revealed a negative correlation between the soil pH before and after biochar application, with the most significant effect observed when the initial soil pH was 3. Biochar has a high ash content, and its application significantly increased the soil pH. Biochar derived from herbaceous materials typically has a higher ash content than biochar from animal sources, resulting in a more pronounced effect on soil pH^39^. Some studies suggest that the higher O% in biochar may be related to its stronger acid‒base buffering capacity, which increases soil pH^40,41^. Additionally, the specific surface area and pore structure of biochar are closely related to its O and H contents. A higher O content typically indicates a better pore structure, which can enhance its CEC and improve its ability to regulate soil pH^42,43^.

### Increases in the CEC and EC of soil due to functional groups on the biochar surface

The results of the current meta-analysis revealed a significant increase in the EC of soil following biochar application (Fig. 1a), with an increase of up to 112% observed when the ash content ranged from 20 to 40%. Previous studies have shown that the application of biochar with a high ash content significantly increases soil EC. Biochar derived from herbaceous materials, which has a higher ash content, exhibits a stronger influence on the EC of soil^44^. Similarly, Brewer et al.(2011) reported that biochar produced from switchgrass and corn stover had a higher ash content than biochar made from hardwood materials^45^, such as red oak and wood waste, at similar temperatures. The carbon in biochar primarily comes from organic material, and a higher C content typically indicates greater stability and durability. Biochar can improve soil structure by providing organic matter to enhance soil moisture retention and reduce salinity, thus influencing the EC^46,47^.

A higher C content in biochar means greater organic matter, which can increase the CEC by providing anionic sites. Biochar with a high C content is generally more stable, and it persists in the soil, continuously contributing to the cation exchange capacity. Consistent with the results of the current meta-analysis, several studies reported that biochar with a higher C content has a greater effect on the CEC of soil^48,49^. The organic residues in biochar created by pyrolysis provide oxygen-containing functional groups (such as carboxyl, carbonyl, and hydroxyl groups), which are related to the O content in biochar^50^. These groups can form coordination bonds with cations, thereby increasing the CEC in soil^51^. In acidic soils, biochar with higher O content can increase the CEC of soil^25^.

### Biochar application and the available Cu and TCLP-extractable Cu contents of soil

Our findings indicate that biochar has a significant effect on available Cu and TCLP-extractable Cu contents, primarily due to its ability to increase soil pH, which in turn promotes a reduction in available Cu and TCLP-extractable Cu contents (Fig. 1a). Increases in soil pH led to significant reductions in the available Cu content, which resulted in a decrease in the TCLP-extractable Cu content (Fig. 1b). The most pronounced effect on the reduction in both available Cu and TCLP-extractable Cu contents occurred when the pH of the biochar was greater than 7.5 (Fig. 6). Previous studies employing XPS and FTIR characterization revealed that the surface of alkaline biochar is rich in functional groups, such as hydroxyl (–OH) and carboxyl (–COOH) groups. These functional groups can form complexes with Cu²⁺ ions, reducing the concentration of bioavailable copper in the soil^52–54^. The impact of biochar on TCLP-extractable Cu content depends on the original soil pH. The dissolved organic matter (DOM) in biochar may form soluble complexes with Cu²⁺ under low pH conditions, thereby increasing the risk of leaching. In contrast, under high pH conditions, these complexes are probably adsorbed onto the biochar surface or precipitated, significantly reducing their leachability^55^. As the original soil pH increased, the effectiveness of biochar in reducing the TCLP-extractable Cu content increased (Fig. 2). Notably, the immobilization of available Cu by biochar was not directly related to the original soil pH (Fig. 2). This may be due to the porous structure of biochar, which can immobilize Cu²⁺ through physical adsorption or micropore trapping. This mechanism primarily depends on surface area and pore distribution rather than pH^56^. However, the concentration of available Cu is negatively correlated with soil pH and decreases with increasing soil pH in both acidic and nonacidic soils (Fig. 4). Biochar treatment effectively regulates soil pH by forming insoluble hydroxide precipitates of Cu ions in the soil, which increases the electronegativity of soil colloids and thus inhibits the biological availability of Cu^57,58^. Moreover, the increases in soil pH lead to increases in negative charges on the soil colloid surface and the soil’s electrostatic effects, thereby enhancing the adsorption capacity of soil colloids for Cu^2+^^12,59^. Our results show that biochar with a higher content of acidic functional groups is more effective in reducing the available Cu and TCLP-extractable Cu contents in acidic soils (Fig. 5)^60,61^. High-polarity biochar, which has a larger surface area^62^promotes the adsorption of Cu ions onto the biochar surface, thereby reducing the concentration of available Cu (Fig. 5b). Additionally, the SSA of biochar facilitates an increase in soil pH (Fig. 5a). Thus, biochar treatment not only enhances soil fertility and provides nutrients necessary for plant growth^44,63^.

The effect of biochar on increasing soil pH was not as significant in nonacidic soils as in acidic soils due to the higher buffering capacity of nonacidic soils. Generally, available Cu levels are relatively low in nonacidic soils^64^. Cu predominantly exists in the form of hydroxides, carbonates, and complexes, thus the application of biochar has a limited impact on available Cu in these soils^65^. High-aromatic biochar is relatively stable and predominantly exists in its oxidized form. Furthermore, the greater the degree of aromatic structure formation, the greater the resistance of biochar to microbial degradation, which reduces the potential for the breakdown of biochar-bound Cu complexes^66–68^. Therefore, the reduction in available Cu contents in nonacidic soils caused by biochar may be due to the adsorption and complexation of organic functional groups^69,70^.

The immobilization of Cu by biochar primarily involves several mechanisms. Precipitation is a primary pathway through which anions released from biochar, such as carbonate and hydroxyl ions, react with Cu^2+^ to form insoluble compounds, such as Cu(OH)₂ and CuCO₃^71^. Surface complexation also plays a crucial role by forming stable coordination complexes between oxygen-containing functional groups (e.g., carboxyl, hydroxyl, and phenolic groups) and Cu^2+^. Furthermore, ion exchange contributes to Cu retention, as Cu^2+^ displaces native cations (e.g., K^+^ and Ca^2+^) adsorbed on the biochar surface^72^. The high surface area and porosity of biochar promote physical adsorption via van der Waals forces and pore-filling effects. Additionally, π–metal interactions between Cu^2+^ and π-electron-rich aromatic domains within the biochar matrix may further enhance Cu immobilization^73^. The biochar ash content, which contains substantial amounts of alkaline substances such as calcium and magnesium, can neutralize acidic ions in the soil and can increase the soil pH^74^. Cu tends to form insoluble compounds, such as Cu hydroxide (Cu(OH)₂), in higher pH environments, thereby reducing the concentration of available Cu^36,75^. In this study, when the O content in the biochar exceeded 20%, the available Cu content was reduced by 22.31% compared to the control group. This might be due to the oxygen-containing functional groups on the biochar surface (such as carboxyl and phenolic hydroxyl groups) forming complexes with Cu ions, thus affecting the amount of available Cu^76,77^. However, some studies suggest that there is a negative correlation between the oxygen content of biochar and its ability to adsorb heavy metals. When the oxygen content of biochar is excessively high, the overabundance of surface oxygen functional groups can reduce its adsorption capacity, particularly for heavy metal ions^78^. The observed discrepancies may be attributed to differences in the feedstock material, pyrolysis temperature, and experimental conditions used in the studies. When biochar has a pH greater than 7.5, the soil pH is significantly increased. In alkaline environments, after the pH of the soil is increased by biochar, water-soluble Cu decreases, leading to a reduction in the TCLP-extractable Cu content^79^. When the H content of the biochar is between 2% and 4%, the TCLP-extractable Cu is significantly reduced by 44.98% in comparison to the control group. This may be due to the relationships between the hydrogen content and the organic matter content, structure, and surface characteristics of the biochar. Additionally, the organic material in biochar can adsorb Cu ions through its porous structure, which reduces the TCLP-extractable Cu content and the leaching, solubility and mobility of Cu^80,81^. Therefore, increasing the pH and O content of biochar during preparation could improve its ability to reduce the available Cu and TCLP-extractable Cu contents of soil.

## Conclusion

This study revealed that variations in the properties of biochar significantly influence its effects on soil chemical properties (pH, CEC, and EC), the available copper (Cu) content, and the Toxicity Characteristic Leaching Procedure (TCLP) extractable Cu content. Biochar application induces complex responses in soil chemistry, leading to increases in soil pH, cation exchange capacity (CEC), and electrical conductivity (EC) by 12.60%, 26%, and 70.30%, respectively, while reducing the available Cu and TCLP-extractable Cu contents by 34.35% and 30.97%, respectively. Biochar increases the pH in both acidic and nonacidic soils, which lowers available Cu and TCLP-extractable Cu levels. The most pronounced pH increase occurs in soils with an initial pH of 3. Biochar with a pH > 7.5 and an oxygen content (O% >20%) demonstrated the greatest efficacy in reducing the available Cu (37.72%) and TCLP-extractable Cu (34.97%) contents, whereas biochar with an ash content > 60% increased the soil pH by 32.4%, and biochar with a specific surface area (SSA) of 50–100 m²/g increased the CEC by 183%. Similarly, biochar with nitrogen (N%) < 2% elevated the EC of soil by 61.27%. Notably, biochar-mediated Cu immobilization does not exhibit a direct relationship with the original soil pH. Therefore, in acidic soils, the application of biochar with a high ash content, high specific surface area, and low nitrogen content (< 2%) can effectively alleviate soil acidification and improve soil quality. Moreover, alkaline biochar with a high oxygen content (O > 20%) and a pH greater than 7.5 is more effective in reducing the availability of Cu in soil. Notably, this meta-analysis did not incorporate soil organic matter (SOM) content or soil type and composition as moderator variables, primarily due to incomplete or inconsistent reporting in the source studies. Although these soil properties are known to significantly influence heavy metal availability, mobility, and retention, particularly under conditions simulated by the TCLP method, relevant data are often absent or reported in incompatible formats. Consequently, our analysis may not fully capture the heterogeneity in Cu extractability arising from differences in SOM content or soil physicochemical characteristics. We recommend that future studies standardize the reporting of these variables to enable more comprehensive and mechanistically informative meta-analyses.

## Supplementary Information

Below is the link to the electronic supplementary material.

Supplementary Material 1.

## Data Availability

No datasets were generated or analysed during the current study.
