# Supplementary material for: Effects of biochar on soil properties as well as available and TCLP-extractable Cu contents: a global meta-analysis
Source: Sci Rep. 2025 Sep 25;15:32853. doi: 10.1038/s41598-025-18170-z (PMC12464268; doi:10.1038/s41598-025-18170-z)
Supplement: Supplementary file 1 — Supplementary Material 1. [file 41598_2025_18170_MOESM1_ESM.docx]

**Supplementary Material**

**Effects of biochar on soil properties, available Cu, and TCLP-extractable Cu: A global meta-analysis**

Xiaowen Teng^a^, Dong Huang^b^, Yaqian Li^c^, Dubin Dong^d^, Xuqiao Wu^a^, Yini Wang^a^, Zhoujia Jiang^a^, Hao Huang^a^, Yanxin Tang^a^, Dan Liu^a*^, Weijie Xu^a**^

^a^ State Key Laboratory of Subtropical Silviculture, Key Laboratory of Soil Remediation and Quality Improvement of Zhejiang Province, Zhejiang A&F University, Lin’an 311300, China.

^b^ Pujiang County Ecological Civilization Promotion Center, Jinhua, 322200, China.

^c^ Agriculture and Rural of Jiaxing, Jiaxing 323500, China.

^d^ College of Life Science and Technology, Central South University of Forestry and Technology, Changsha 410004, China.

**^*^Corresponding authors:**

Dan Liu; E-mail: [liudan@zafu.edu.cn](mailto:liudan@zafu.edu.cn)

Weijie Xu; E-mail: [xuweijie@zafu.edu.cn](mailto:liudan@zafu.edu.cn)

**Table S1** Results of publication bias about datasets in this study. “*N*” is the number of observations.

| **Factors** | **Soil pH** | **EC** | **CEC** | **Available Cu** | **TCLP Cu** |
| --- | --- | --- | --- | --- | --- |
| Observations sizes (N) | 189 | 86 | 32 | 223 | 63 |
| 5*N*+10 | 955 | 440 | 170 | 1125 | 325 |
| Fail-safe number | 7700288 | 1459157 | 39342 | 1980635 | 426074 |
| Target significance level | 0.05 | 0.05 | 0.05 | 0.05 | 0.05 |
| Publication bias | No | No | No | No | No |

**Figure captions**

**Fig. S1.** Flow diagram of peer-reviewed papers selection for the meta-analysis.

**Fig. S2.** Distribution of the studies selected in the meta-analysis.


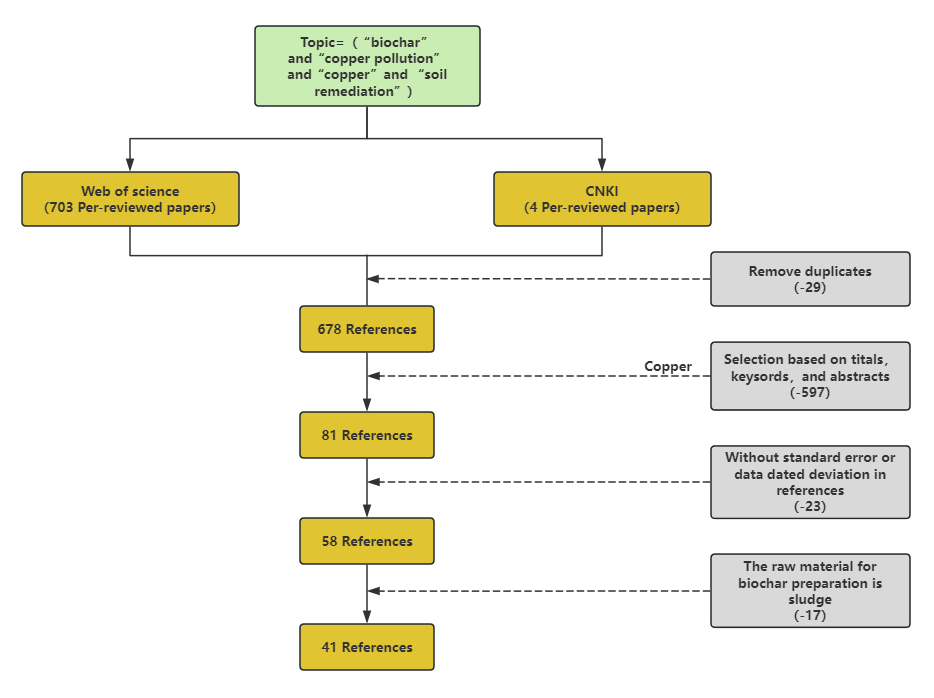


**Fig. S1.** Flow diagram of peer-reviewed papers selection for the meta-analysis.


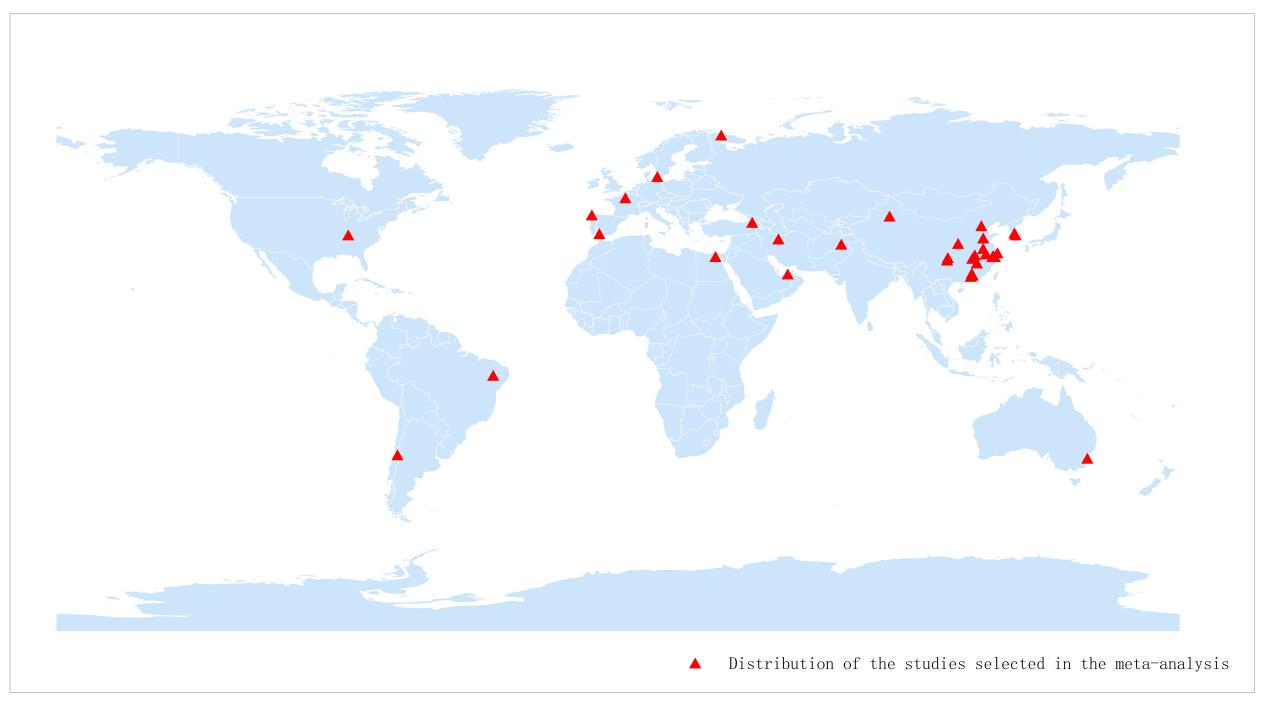


**Fig. S2.** Distribution of the studies selected in the meta-analysis.
